# Prognostic Significance of Lymph Node Yield on Disease-Free Survival and Overall Survival in Patients With Small Bowel Neuroendocrine Tumors

**DOI:** 10.7759/cureus.86896

**Published:** 2025-06-27

**Authors:** Muhammad S Naeem, Muhammad Zubair, Sana Ejaz, Nida Saleem, Zain Tayyab, Muhammad Usama, Muhammad A Riaz, Rabbya Naseem, Aun Jamal, Aamir Syed, Hafiza S Ramzan

**Affiliations:** 1 Surgery, Mayo Hospital, Lahore, PAK; 2 Surgical Oncology, Shaukat Khanum Memorial Cancer Hospital and Research Centre, Lahore, PAK; 3 Breast Surgery, Shaukat Khanum Memorial Cancer Hospital and Research Centre, Lahore, PAK; 4 Medicine, Services Hospital Lahore, Lahore, PAK; 5 Biochemistry, Institute of Biochemistry and Biotechnology, Lahore, PAK

**Keywords:** bowel disorder, cancer patients, patients satisfaction, prognostic tools, progression-free survival

## Abstract

Background

Small bowel neuroendocrine tumors (SB-NETs) are increasingly recognized gastrointestinal neoplasms with distinct biological behavior and a high tendency for lymphatic spread. Accurate pathological staging, particularly lymph node yield (LNY), plays a pivotal role in determining prognosis and guiding treatment strategies.

Objective

To evaluate the prognostic significance of lymph node yield and other clinicopathological features in relation to disease-free and overall survival in patients with SB-NETs. Methods: This retrospective study included 62 patients with histologically confirmed SB-NETs treated at a tertiary care center. Demographic data, tumor characteristics, Ki-67 index, presence of metastases, type of surgery, and adjuvant treatment were recorded.

Results

The mean age at diagnosis was 51.9 ± 10.2 years. There was a male predominance, with 44 (71.0%) patients being male. The most common presenting symptom was abdominal pain, reported in 31 (50.0%) patients. Lymph node metastases were present in 32 (51.6%) patients, while liver metastases were observed in 15 (24.2%) patients. The most frequent primary tumor site was the duodenum, found in 28 (45.2%) patients. A low Ki-67 index (≤2%) was observed in 27 (43.5%) cases. Surgical resection was performed in the majority, with laparotomy being the most common approach in 36 (58.1%) patients. Adjuvant therapy was administered to 20 (32.3%) patients.

Conclusion

It is concluded that lymph node yield is a key prognostic factor in SB-NETs and should be emphasized in surgical planning. Nodal and liver metastases are common at presentation, underscoring the need for comprehensive staging.

## Introduction

Small bowel neuroendocrine tumors (SB-NETs) are a type of gastrointestinal neuroendocrine tumor originating from the serotonin-secreting enterochromaffin cells of the intestine [1]. The small intestine is the most common site for these tumors in the gastrointestinal tract, accounting for approximately 30% of cases, followed by the rectum at 19% [2]. Representing nearly 48% of small bowel neoplasms, SB-NETs are the most frequent malignancy in this part of the gastrointestinal tract [3]. Despite their relatively slow growth, SB-NETs often present a diagnostic challenge due to their nonspecific symptoms and potential for metastatic spread at the time of diagnosis [4]. Survival outcomes for patients with SB-NETs vary widely and are influenced by factors such as tumor stage, primary tumor site, race, and age at diagnosis [5]. The median overall survival for all neuroendocrine tumors is approximately 9.3 years [6]. One of the critical areas of ongoing research and debate is the management of lymph node involvement in SB-NETs [7]. The extent of lymph node dissection required for optimal staging and therapeutic benefit remains unclear, particularly in cases of localized versus advanced locoregional disease with mesenteric nodal involvement. Surgical resection remains the cornerstone of curative-intent treatment for SB-NETs. According to the European Neuroendocrine Tumor Society (ENETS) and the North American Neuroendocrine Tumor Society (NANETS), the standard surgical approach includes segmental resection of the involved bowel with en bloc mesenteric lymphadenectomy. However, the optimal extent of lymph node dissection and its impact on long-term outcomes remain subjects of ongoing debate [8]. While radical resections that include mesenteric lymph nodes are encouraged, the variability in lymph node yields across institutions and surgeons leads to inconsistent pathological staging and potentially suboptimal prognostication [9].

Lymph node yield (LNY) refers to the total number of lymph nodes retrieved and evaluated during oncologic surgery. In several solid tumors, including colorectal, gastric, and pancreatic cancers, higher LNY has been consistently associated with improved survival outcomes. This is hypothesized to result from more accurate staging, better removal of micrometastatic disease, and a reflection of the host immune response or tumor biology [9]. Despite the biological rationale and preliminary clinical support, there is a lack of consensus regarding the minimum number of lymph nodes that should be harvested for SB-NETs to provide adequate staging and prognostic utility. Some studies suggest a threshold of ≥8 or ≥12 lymph nodes as being associated with improved survival, yet these findings are inconsistent and largely based on retrospective analyses with heterogeneous cohorts [1]. Moreover, unlike colorectal cancer, where such thresholds are widely accepted and embedded into clinical guidelines, SB-NETs lack standardized lymphadenectomy guidelines, in part due to their rarity and the absence of large prospective datasets [2]. The prognostic relevance of LNY in SB-NETs also intersects with the issue of mesenteric fibrosis, a frequent complication associated with serotonin-secreting tumors, which can obscure nodal planes, making dissection more challenging and sometimes limiting the total nodes retrieved. Therefore, lymph node yield may not only reflect surgical diligence but also capture an element of tumor-induced desmoplastic reaction, further complicating its interpretation [10].

Objectives

The objective of this study was to evaluate the prognostic significance of lymph node yield in small bowel neuroendocrine tumors and to determine the optimal number of lymph nodes required for accurate staging and improved patient outcomes.

## Materials and methods

This retrospective cross-sectional study was conducted at Shaukat Khanum Memorial Cancer Hospital and Research Center from January 2014 to December 2023.

Inclusion and exclusion criteria

Inclusion criteria were patients with a confirmed histopathological diagnosis of SB-NETs who underwent segmental small bowel resection and regional lymphadenectomy. The exclusion criteria included patients who were lost to follow-up, had limited patient data available, contraindication to surgery due to severe comorbidities, inoperable disease on scans, and no radiological evidence of loco-regional disease.

Data collection

Data were collected from the hospital's electronic medical records and pathology archives. Information regarding patient demographics, tumor characteristics (location, size, histological grade, Ki-67 index), surgical details (extent of resection, number of lymph nodes retrieved), margin status, and presence of mesenteric fibrosis was obtained. The total number of lymph nodes harvested and the number of metastatic nodes were recorded for each patient. Patients were stratified into two groups based on lymph node yield using a threshold value determined by literature review and the Receiver Operating Characteristic (ROC) curve analysis (e.g., ≥8 vs. <8 lymph nodes). Survival outcomes were compared between these groups. Patients were followed every three to six months postoperatively with physical examination, biochemical markers (including chromogranin A and 5-hydroxyindoleacetic acid (5-HIAA) when applicable), and imaging studies (CT/MRI and/or gallium-68 DOTATATE positron emission tomography (PET) scan). Follow-up data were censored at the time of last contact or death.

Statistical analysis

Data were analyzed using SPSS version 26.0 (IBM Inc., Armonk, New York). Continuous variables were presented as mean ± standard deviation or median (IQR), depending on distribution. Categorical variables were presented as frequencies and percentages. A p-value of <0.05 was considered statistically significant.

## Results

Data were collected from 62 patients. The mean age at diagnosis was 51.9 ± 10.2 years, with a male predominance of 44 (71.0%). Carcinoid symptoms were observed in nine (14.5%) patients, while liver metastases were present in 15 (24.2%). The primary tumor site was most commonly the duodenum in 28 (45.2%) patients, followed by the ileum in 20 (32.3%) and the jejunum in 10 (16.1%). Lymph node metastases were detected in 32 (51.6%) patients. The most frequently observed pathological stage groups were T2N1-T3N0 in 13 (20.9%) and T3N1 in 11 (17.7%), indicating a substantial burden of nodal involvement even in intermediate-stage disease (Table 1).

**Table 1:** Baseline demographics and tumor characteristics

Variable	N	Percent (%)
Total patients	62	100.0
Gender - male	44	71.0
Gender - female	14	22.6
Mean age at diagnosis (years)	51.9 ± 10.2	-
Carcinoid symptoms - yes	9	14.5
Carcinoid symptoms - no	49	79.0
Liver metastases - yes	15	24.2
Liver metastases - no	43	69.4
Primary site - duodenum	28	45.2
Primary site - jejunum	10	16.1
Primary site - ileum	20	32.3
Lymph node metastases - present	32	51.6
Lymph node metastases - absent	24	38.7
Stage group		
T1-T2N0	4	6.4
T2N1-T3N0	13	20.9
T3N1	11	17.7
T3N1M1-T3N2	5	8.0
T4 stages	10	16.1

Nearly half of the patients, 30 (48.4%), had no reported comorbidities at the time of diagnosis. Among those with comorbid conditions, hypertension was the most prevalent, affecting 12 (19.4%) patients, followed by diabetes mellitus in six (9.7%) patients. Less commonly reported conditions included hepatitis C in four (6.5%), hypothyroidism in two (3.2%), and chronic kidney disease in one (1.6%) patient. A small proportion of patients also had ischemic heart disease (1, 1.6%) or other unspecified comorbidities (Table 2).

**Table 2:** Comorbidities distribution

Comorbidity	N	Percent (%)
Hypertension	12	19.4
Diabetes	6	9.7
Ischemic heart disease	1	1.6
Hepatitis C	4	6.5
Hypothyroidism	2	3.2
Chronic kidney disease	1	1.6
Others	2	3.2
None	30	48.4

Abdominal pain was the most frequently reported symptom, present in 31 (50.0%) patients, followed by malena and epigastric pain, each observed in nine (14.5%) patients. A smaller number of patients experienced obstruction in five (8.1%) and diarrhea in three (4.8%), while only one (1.6%) patient reported other non-specific symptoms. Regarding post-operative management, 20 (32.3%) patients received adjuvant treatment, whereas the majority, 38 (61.3%), did not, reflecting variability in clinical staging and risk stratification (Table 3).

**Table 3:** Clinical presentation

Symptom	N	Percent (%)
Abdominal pain	31	50.0
Diarrhea	3	4.8
Malena	9	14.5
Obstruction	5	8.1
Epigastric pain	9	14.5
Others	1	1.6
Adjuvant treatment		
Yes	20	32.3
None	38	61.3

The Ki-67 proliferation index, an important marker of tumor aggressiveness, was ≤2% in 27 (43.5%) patients, with the majority falling in the 1-2% range, specifically 19 (30.6%), indicating a predominance of well-differentiated, slow-growing tumors. Only five (8.1%) patients had a Ki-67 index >20%, suggestive of more aggressive disease. The surgical approach was primarily open laparotomy in 36 (58.1%) patients, while laparoscopic procedures were performed in four (6.5%), and 13 (21.0%) patients underwent no surgical intervention. The most common site of the primary tumor was the duodenum in 28 (45.2%) patients, followed by the ileum in 20 (32.3%) and the jejunum in 10 (16.1%) (Table 4).

**Table 4:** Ki-67 proliferation index

Ki-67 index group	N	Percent (%)
<1%	8	12.9
1–2%	19	30.6
3–5%	6	9.7
6–20%	11	17.7
>20%	5	8.1
Mode of surgery		
Laparotomy	36	58.1
Laparoscopic	4	6.5
Others	5	8.1
None	13	21.0
Primary tumor site		
Duodenum	28	45.2
Jejunum	10	16.1
Ileum	20	32.3

Lymph node metastasis was identified in 32 (51.6%) patients, highlighting a high rate of regional nodal involvement even in small bowel neuroendocrine tumors. In contrast, liver metastases were observed in 15 (24.2%) patients, while 43 (69.4%) had no evidence of hepatic spread at diagnosis (Table 5).

**Table 5:** Lymph node metastasis

Lymph node metastasis	N	Percent (%)
Present	32	51.6
Absent	24	38.7
Liver metastasis		
Present	15	24.2
Absent	43	69.4

## Discussion

This study aimed to evaluate the clinicopathological features and prognostic markers in patients diagnosed with small bowel neuroendocrine tumors (SB-NETs), with a specific focus on lymph node and liver metastases, Ki-67 proliferation index, and treatment outcomes. The analysis of 62 patients revealed several key insights into disease presentation, progression, and treatment patterns in a tertiary care setting. The majority of patients were male with a mean age of 51.9 years, which is consistent with global epidemiological trends showing a slight male predominance and a peak incidence in the fifth to sixth decade of life. Abdominal pain emerged as the most common presenting symptom, followed by malena and epigastric discomfort. Carcinoid syndrome was documented in a relatively small subset (14.5%), in line with literature suggesting that classic hormonal symptoms occur more frequently in cases with advanced or metastatic disease [11].

A significant proportion of patients (51.6%) exhibited lymph node metastases, emphasizing the critical role of thorough nodal evaluation during surgical management. Lymph node yield has been associated with both accurate staging and improved survival in gastrointestinal cancers, and its significance in SB-NETs continues to be supported by recent studies [12]. In this cohort, liver metastases were present in 24.2% of patients, which is slightly lower than the 30-40% typically reported in Western populations, possibly reflecting earlier detection or selection bias. The Ki-67 proliferation index was ≤2% in the majority of patients, suggesting well-differentiated tumor biology and generally favorable prognosis. However, the presence of high-grade tumors (>20% Ki-67) in a minority of patients highlights the heterogeneity within this disease spectrum and the need for individualized therapeutic strategies [13]. Surgical resection, primarily through laparotomy, was performed in most cases, reinforcing its role as the cornerstone of curative management. A smaller number of patients underwent laparoscopic procedures or were managed conservatively, often due to advanced disease or comorbid conditions [14]. The predominance of grade 1 tumors in this cohort aligns with findings from Sorbye et al. (2013), who reported that nearly half of SB-NETs in their multicenter study fell into the low-grade category [15]. However, a subset (8.1%) in our study had a Ki-67 index >20%, which is classified as grade 3 and suggests a more aggressive clinical course, warranting closer monitoring or systemic therapy [16]. Interestingly, nearly half of the patients had no reported comorbidities, which could have contributed to the higher rate of surgical intervention. Among those with comorbid conditions, hypertension and diabetes were the most frequent. Adjuvant therapy was administered in 32.3% of patients, typically in those with high-risk pathological features, although clear guidelines on adjuvant treatment in SB-NETs are still lacking, and practice remains variable. This study has several limitations that must be acknowledged. First, its retrospective design inherently introduces selection bias and limits the ability to establish causal relationships. The relatively small sample size from a single tertiary care center may affect the generalizability of the findings to broader populations. Additionally, the absence of uniform criteria for administering adjuvant therapy and the variability in follow-up duration may have influenced survival outcomes.

## Conclusions

It is concluded that small bowel neuroendocrine tumors (SB-NETs) exhibit a high rate of lymph node involvement at the time of diagnosis, making lymph node yield a critical factor for accurate staging and prognostic assessment. The presence of liver metastases and higher Ki-67 proliferation index further correlates with advanced disease and underscores the heterogeneity within this tumor group. Surgical resection with adequate lymphadenectomy remains the cornerstone of management, while the role of adjuvant therapy should be personalized based on individual risk profiles.

## References

[1] Yao JC, Hassan M, Phan A (2008). One hundred years after "carcinoid": epidemiology of and prognostic factors for neuroendocrine tumors in 35,825 cases in the United States. J Clin Oncol.

[2] Pinchot SN, Holen K, Sippel RS, Chen H (2008). Carcinoid tumors. Oncologist.

[3] Anzidei M, Napoli A, Zini C, Kirchin MA, Catalano C, Passariello R (2011). Malignant tumours of the small intestine: a review of histopathology, multidetector CT and MRI aspects. Br J Radiol.

[4] Forero Molina MA, Garcia E, Gonzalez-Devia D, García-Duperly R, Vera A (2017). A 17-year-old male with a small bowel neuroendocrine tumor: flushing differential diagnosis. World Allergy Organ J.

[5] Liu Y, Ye S, Zhu Y (2019). Impact of tumour size on metastasis and survival in patients with pancreatic neuroendocrine tumours (PNETs): a population based study. J Cancer.

[6] Dasari A, Shen C, Halperin D (2017). Trends in the incidence, prevalence, and survival outcomes in patients with neuroendocrine tumors in the United States. JAMA Oncol.

[7] Ferris RL, Lotze MT, Leong SP, Hoon DS, Morton DL (2012). Lymphatics, lymph nodes and the immune system: barriers and gateways for cancer spread. Clin Exp Metastasis.

[8] Landry CS, Lin HY, Phan A (2013). Resection of at-risk mesenteric lymph nodes is associated with improved survival in patients with small bowel neuroendocrine tumors. World J Surg.

[9] Zaidi MY, Lopez-Aguiar AG, Dillhoff M (2019). Prognostic role of lymph node positivity and number of lymph nodes needed for accurately staging small-bowel neuroendocrine tumors. JAMA Surg.

[10] Grillo F, Albertelli M, Malandrino P (2022). Prognostic effect of lymph node metastases and mesenteric deposits in neuroendocrine tumors of the small bowel. J Clin Endocrinol Metab.

[11] Kroepfl V, Bellotti R, Gasser E (2023). Small bowel neuroendocrine tumors: an analysis of clinical presentation, diagnostic workup and surgical approach - a single center retrospective study. Front Surg.

[12] Wong W, Perez Holguin RA, Olecki EJ (2022). Predictors and outcomes of minimally invasive surgery for small bowel neuroendocrine tumors: minimally invasive surgery for SBNETs. J Gastrointest Surg.

[13] Tran CG, Sherman SK, Howe JR (2020). Small bowel neuroendocrine tumors. Curr Probl Surg.

[14] Clift AK, Drymousis P, von Roon A (2023). Management of small bowel neuroendocrine tumours: 10 years' experience at a tertiary referral centre. Cancers (Basel).

[15] Sorbye H, Welin S, Langer SW (2013). Predictive and prognostic factors for treatment and survival in 305 patients with advanced gastrointestinal neuroendocrine carcinoma (WHO G3): the NORDIC NEC study. Ann Oncol.

[16] Pavel M, Öberg K, Falconi M, Krenning EP, Sundin A, Perren A, Berruti A (2020). Gastroenteropancreatic neuroendocrine neoplasms: ESMO Clinical Practice Guidelines for diagnosis, treatment and follow-up. Ann Oncol.

